# Tracking Protests Using Geotagged *Flickr* Photographs

**DOI:** 10.1371/journal.pone.0150466

**Published:** 2016-03-01

**Authors:** Merve Alanyali, Tobias Preis, Helen Susannah Moat

**Affiliations:** Data Science Lab, Behavioural Science, Warwick Business School, University of Warwick, Coventry, CV4 7AL, United Kingdom; IFIMAR, UNMdP-CONICET, ARGENTINA

## Abstract

Recent years have witnessed waves of protests sweeping across countries and continents, in some cases resulting in political and governmental change. Much media attention has been focused on the increasing usage of social media to coordinate and provide instantly available reports on these protests. Here, we investigate whether it is possible to identify protest outbreaks through quantitative analysis of activity on the photo sharing site *Flickr*. We analyse 25 million photos uploaded to *Flickr* in 2013 across 244 countries and regions, and determine for each week in each country and region what proportion of the photographs are tagged with the word “protest” in 34 different languages. We find that higher proportions of “protest”-tagged photographs in a given country and region in a given week correspond to greater numbers of reports of protests in that country and region and week in the newspaper *The Guardian*. Our findings underline the potential value of photographs uploaded to the Internet as a source of global, cheap and rapidly available measurements of human behaviour in the real world.

## Introduction

Smartphones and computers are becoming an indispensible part of everyday life in many countries around the globe. Usage of these devices and the online services they connect us to is generating fast and cheap measurements of human behaviour at a global scale. Research in the growing interdisciplinary field of computational social science [1–7] has begun to draw on this rich new data source, exploiting data from search engines such as *Google* [8–13] and *Yahoo* [14, 15], the online encyclopedia *Wikipedia* [16, 17], news sources such as the *Financial Times* [18] as well as social media platforms including *Twitter* [19–22] and the photo sharing website *Flickr* [23–26]. Studies to date have demonstrated that appropriate analyses of these online datasets can offer estimates of key economic and health indicators before official figures are released [10, 27–30] and in some cases, improve forecasts of real world economic decision making [13–15, 17–19].

In recent years, news reports have described a number of prominent outbursts of protests in countries around the world, in some cases leading to political change. Much media attention has been focused on the increasing usage of social media to coordinate and provide instantly available reports on these protests [31–33]. As a result of improved connectivity, posts to social media sites are steadily beginning to shift from solely text based reports to sharing of visual media such as photographs and videos. Here, we explore whether the data created through such widespread usage of online services may offer a valuable new source of measurements of behaviour during protests. Specifically, we investigate whether data on photographs uploaded to the photo sharing website *Flickr* can be used to identify protest outbreaks around the world.

## Materials and Methods

We analyse a large corpus of metadata on the 24,944,764 geotagged photographs taken and uploaded to *Flickr* between 1^st^ January 2013 and 31^st^ December 2013. We retrieved data on image uploads to *Flickr* by accessing the *Flickr* API in January 2014, and downloading data in JSON format using R 3.0.1. The metadata we analyse comprise a wide range of information on where and when a photograph was taken, information about the photographer, as well as user chosen title, description and tags for each photograph, and the URL from which the photograph can be downloaded.

For each geotagged photograph, we retrieve data on both the time and the place at which the photograph was taken. For each week, for each of the 242 countries and regions listed in S1 Table, as well as the United Kingdom and the United States, we determine how many photographs were taken and uploaded with the word “protest” in English in either the title, photograph description or photograph tag. We also translate the word “protest” into 33 further languages, by accessing the “Protest” article on the English language *Wikipedia*, and using the title of all articles on versions of *Wikipedia* which are not in English, but which are linked as translations of the article. The complete list of translations is provided in S2 Table. The counts of photographs taken and shared on *Flickr* throughout 2013 in each of the 244 countries and regions are listed in S3 Table.

The overall number of photos taken and uploaded to *Flickr* in different countries and regions may differ. To account for this, we extract the total number of photos taken and uploaded during each week in 2013 for each of the 244 countries and regions analysed. We consider a week as starting on a Monday and ending on a Sunday. Using these counts, we normalise the weekly counts of photographs taken in each country and region in each week, by dividing the number of photographs labelled with a word signifying “protest” by the weekly count of all photos taken in the same country and region.

To determine whether we can find any evidence that changes in the number of protest-tagged photographs taken and uploaded to *Flickr* correspond to changes in the number of protest outbreaks, we require data on when and where protests have occurred. Such ground truth data can be difficult to obtain. Most studies of civil unrest therefore rely on data from newspaper reports as a proxy for ground truth [22, 34–36]. Following this approach, here we determine how many protest related articles for each of the 244 countries and regions were published in the online edition of *The Guardian* in each week in 2013.

We retrieved data on articles in the online edition of *The Guardian* via *The Guardian* Developer Toolbox in January 2016. We deem an article as protest related if it is tagged with the word “protest”, and we deem an article as covering news related to one of the 244 countries and regions analysed if it is tagged with the country and region’s name. To account for differences in coverage of news in different countries and regions by *The Guardian*, we also determine the total number of articles published in each week and tagged with each country’s name. In total, we analyse data on 178,730 articles from *The Guardian*. The counts of *The Guardian* articles published in 2013 and tagged with each of the of the country and region names are listed in S4 Table. We note that *The Guardian* uses a different tagging system for articles relating to the United Kingdom and the United States. For this reason, we determine the number of articles relating to the United Kingdom by counting articles tagged with the names “England”, “Scotland”, “Wales” and “Northern Ireland”, and we determine the number of articles relating to the United States by counting articles tagged “US news”.

In order to model the relationship between *Flickr* user activity and protest outbreaks, we build a logistic regression panel model. The outcome variable is whether a *The Guardian* article is protest related or not. To control for underlying differences in the number of protests in a given country and region and week, we include country and region and week as fixed effects in our model. We underline that in this analysis, we focus on the relationship between *Flickr* activity and protest reports within the same week. Future analyses may wish to investigate whether photographic data can be used to predict protest activity before it occurs.

## Results

**Fig 1:**
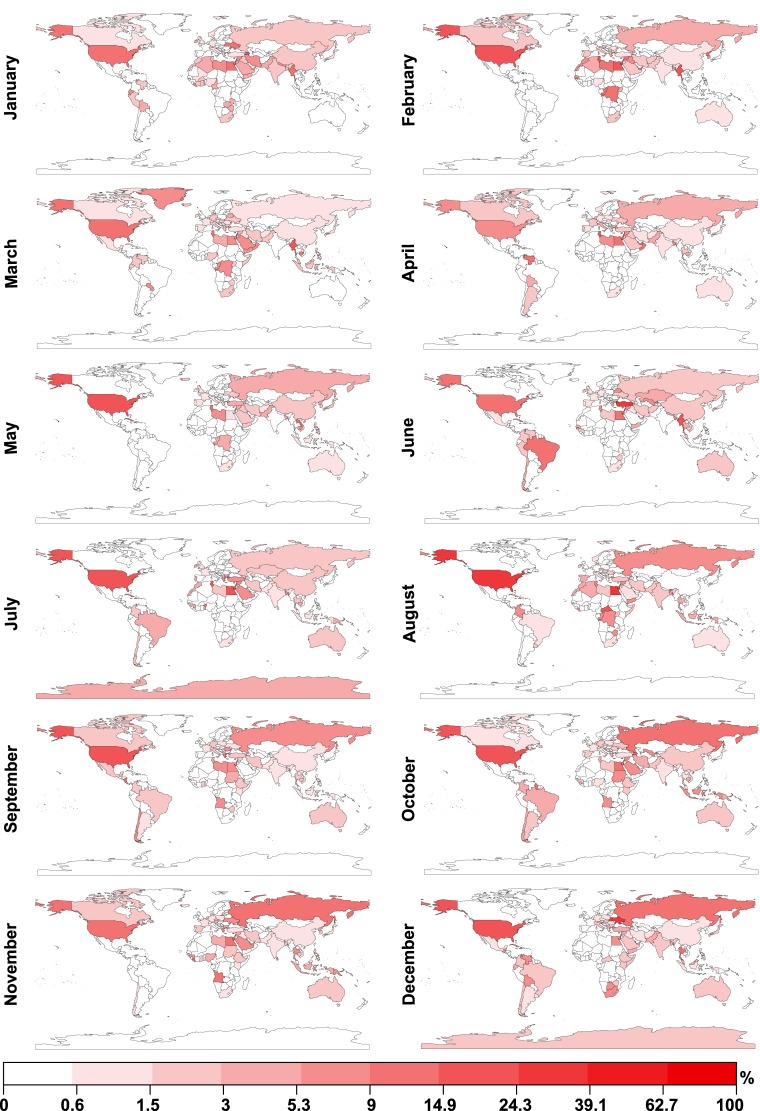
Reports of protests in 2013 in the online edition of *The Guardian*. We use data on reports of protests in the online edition of *The Guardian* as an approximation of the ground truth of when and where notable protest outbreaks occurred. For each of the 244 countries and regions for each month in 2013, we calculate the number of *The Guardian* articles tagged with the country and region’s name. Here, we depict the percentage of articles for each country and region and each month which were also tagged with the word “protest”. Patterns which can be visually identified in the data reflect known major protest events in 2013: for example, protest outbreaks in both Brazil and Turkey can be observed in June 2013. Equal breaks are calculated for the logarithmically transformed percentages.

We use data on reports of protests in the online edition of *The Guardian* as an approximation of the ground truth of when and where protest outbreaks occurred. For each of the 244 countries and regions analysed, for each month in 2013, we calculate the number of *The Guardian* articles tagged with the country and region’s name. In Fig 1, we depict the percentage of articles for each country and region and each month which were also tagged with the word “protest”. Patterns which can be visually identified in the data reflect known major protest events in 2013: for example, protest outbreaks in both Brazil and Turkey can be observed in June 2013.

**Fig 2:**
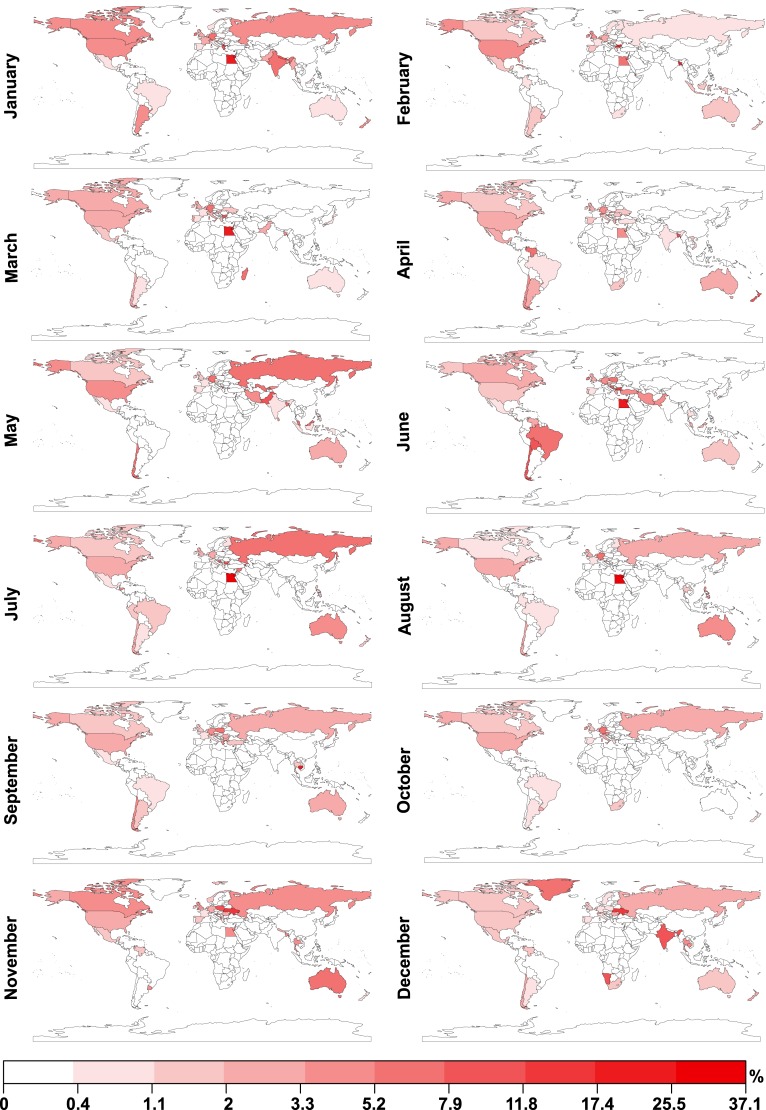
Locations of *Flickr* photographs labelled with “protest” in 2013. We investigate to what extent data on the number of photographs tagged with the word “protest” and uploaded to *Flickr* reflect the ground truth data extracted from *The Guardian*. For each of the 244 countries and regions for each month in 2013, we calculate the total number of geotagged photographs taken and uploaded to *Flickr*. Here, we visualise the percentage of photographs for each country and region and each month which were also labelled with the character sequence “protest”. Visual inspection suggests that while there are clear differences between the spatio-temporal distributions of “protest” labelled *Flickr* photographs and “protest” labelled articles in *The Guardian*, some key similarities can also be identified, such as an increase in “protest” labelled *Flickr* photographs in Brazil and Turkey in June 2013. Equal breaks are calculated for the logarithmically transformed percentages.

We examine to which extent data on the number of photographs tagged with the word “protest” and uploaded to *Flickr* reflect the ground truth data extracted from *The Guardian*. Again, for each of the 244 countries and regions listed in S1 Table, for each month in 2013, we calculate the total number of geotagged photographs taken and uploaded to *Flickr*. In Fig 2, we visualise the percentage of photographs for each country and region and each month which were also labelled with a word signifying “protest” in one of the 34 languages identified above and listed in S2 Table. Visual inspection suggests that while there are clear differences between the spatio-temporal distributions of “protest” labelled *Flickr* photographs and “protest” labelled articles in *The Guardian*, some key similarities can also be identified, such as an increase in “protest” labelled *Flickr* photographs in Brazil and Turkey in June 2013.

To determine whether we can find statistical evidence of a relationship between the number of “protest” labelled photographs taken and uploaded to *Flickr* and reports of protests in *The Guardian*, we consider both datasets at weekly granularity. For each week in 2013, for each country and region, we calculate the number of geotagged photographs taken and uploaded to *Flickr* which are labelled with the character sequence “protest” in 34 different languages, and normalise this count by the total number of geotagged photographs taken and uploaded to *Flickr* in that week and country and region. To analyse the relationship between the data mined from *Flickr* and reports of protests in *The Guardian*, we build a logistic regression panel model. To account for unobserved differences in coverage between countries and regions and weeks, we include country and region and week as fixed effects.

Our results suggest that a greater normalised number of “protest” labelled *Flickr* photographs in a given week and country and region corresponds to a greater proportion of *The Guardian* articles about that country and region being tagged with the word “protest” (*Flickr* predictor: *β* = 2.95, *SE* = 0.31, *z* = 9.48, *N* = 12932, *p* < 0.001). The odds ratio corresponding to an increase of 0.1 in the normalised number of “protest” tagged *Flickr* photos is 1.34 (calculated using *β* and the value of the logistic regression intercept, -5.09). This implies that if we fix the country and region and week effects, increasing the normalised number of “protest” tagged *Flickr* pictures by 0.1 will increase the odds of a protest related *The Guardian* article by 34%.

For comparison, we construct a simple baseline model which captures differences in protest frequency between countries and regions, and differences in protest frequencies across different weeks, by building a logistic regression panel model with country and region and week as fixed effects, leaving out the *Flickr* predictor. We find that the model including data on the normalised number of “protest” labelled *Flickr* photographs allows us to account for more variance in the proportion of *The Guardian* articles tagged with the word “protest” than this simple baseline model of differences between different countries and regions and different weeks (*McFadden* *R*^2^ for baseline model = 0.34, *McFadden* *R*^2^ for *Flickr* model = 0.35, *χ*^2^(1) = 84.48, *p* < 0.001, Likelihood Ratio Test).

In line with other studies of civil unrest, our analysis uses data from newspaper reports of protests as a proxy for ground truth data on protest occurrences [34–36]. As a result, we cannot rule out the possibility that *Flickr* users are posting photographs labelled with a word signifying “protest” as a result of reading an article about protests in their country and region in *The Guardian*, or another news source. We posit however that the geotagged nature of the *Flickr* photographs we analyse makes it less likely that such an explanation may hold, in contrast with simple time series analyses of online behaviour on services such as *Google* or *Twitter*, where searching behaviour or tweets may reflect reactions to news articles. With this caveat in mind, our results are consistent with the hypothesis that data on photographs posted to *Flickr* may help us to identify protest outbreaks around the world.

## Discussion

We investigate whether data on photographs uploaded to the photo sharing website *Flickr* may be of use in identifying protest outbreaks. We analyse 25 million photos uploaded to *Flickr* in 2013 across 244 countries and regions, and determine for each week in each country and region what proportion of the photographs are tagged with the word “protest” in 34 different languages. We find that higher proportions of “protest”-tagged photographs in a given country and region in a given week correspond to greater numbers of reports of protests in that country and region and week in the newspaper *The Guardian*. These results are in line with the striking hypothesis that data on photographs uploaded to *Flickr* may contain signs of protest outbreaks. Our findings underline the potential value of photographs uploaded to the Internet as a source of global, cheap and rapidly available measurements of human behaviour in the real world.

## Supporting Information

S1 Table Country and region names. List of country and region names used in analysis.
(PDF)

S2 Table Translations of “protest”. List of translations of the word “protest” in different languages.
(PDF)

S3 Table Flickr photograph counts per country and region in 2013. Total number of photographs per country and region taken and uploaded to *Flickr* during 2013.
(PDF)

S4 Table Counts of articles in The Guardian per country and region in 2013. Total number of *The Guardian* articles released during 2013 covering news relating to countries and regions listed.
(PDF)
