# Supplementary material for: Tracking Protests Using Geotagged Flickr Photographs
Source: PLoS One. 2016 Mar 1;11(3):e0150466. doi: 10.1371/journal.pone.0150466 (PMC4773018; doi:10.1371/journal.pone.0150466)
Supplement: S1 Table — List of country and region names used in analysis. (PDF) [file pone.0150466.s001.pdf]

S1 Table. List of the country and region names as used in the analyses.

Afghanistan	Aland	Albania
Algeria	American Samoa	Andorra
Angola	Anguilla	Antarctica
Antigua and Barbuda	Argentina	Armenia
Aruba	Ashmore and Cartier Islands	Australia
Austria	Azerbaijan	Bahrain
Bangladesh	Barbados	Belarus
Belgium	Belize	Benin
Bermuda	Bhutan	Bolivia
Bosnia and Herzegovina	Botswana	Brazil
British Indian Ocean Territory	British Virgin Islands	Brunei
Bulgaria	Burkina Faso	Burundi
Cambodia	Cameroon	Canada
Cape Verde	Cayman Islands	Central African Republic
Chad	Chile	China
Colombia	Comoros	Cook Islands
Costa Rica	Croatia	Cuba
Curacao	Cyprus	Czech Republic
Democratic Republic of the Congo	Denmark	Djibouti
Dominica	Dominican Republic	East Timor
Ecuador	Egypt	El Salvador
Equatorial Guinea	Eritrea	Estonia
Ethiopia	Falkland Islands	Faroe Islands
Federated States of Micronesia	Fiji	Finland
France	French Guiana	French Polynesia
French Southern and Antarctic Lands	Gabon	Gambia
Gaza	Georgia	Germany
Ghana	Greece	Greenland
Grenada	Guam	Guatemala
Guernsey	Guinea	Guinea Bissau
Guyana	Haiti	Heard Island and McDonald Islands
Honduras	Hong Kong S.A.R.	Hungary
Iceland	India	Indian Ocean Territories
Indonesia	Iran	Iraq
Ireland	Isle of Man	Israel
Italy	Ivory Coast	Jamaica
Japan	Jersey	Jordan
Kazakhstan	Kenya	Kiribati
Kosovo	Kuwait	Kyrgyzstan
Laos	Latvia	Lebanon
Lesotho	Liberia	Libya
Liechtenstein	Lithuania	Luxembourg
Macau S.A.R	Macedonia	Madagascar
Malawi	Malaysia	Maldives
Mali	Malta	Marshall Islands
Mauritania	Mauritius	Mexico
Moldova	Monaco	Mongolia
Montenegro	Montserrat	Morocco
Mozambique	Myanmar	Namibia

Nauru	Nepal	Netherlands
New Caledonia	New Zealand	Nicaragua
Niger	Nigeria	Niue
Norfolk Island	Northern Cyprus	Northern Mariana Islands
North Korea	Norway	Oman
Pakistan	Palau	Panama
Papua New Guinea	Paraguay	Peru
Philippines	Pitcairn Islands	Poland
Portugal	Puerto Rico	Qatar
Republic of Serbia	Republic of the Congo	Romania
Russia	Rwanda	Saint Barthelemy
Saint Helena	Saint Kitts and Nevis	Saint Lucia
Saint Martin	Saint Pierre and Miquelon	Saint Vincent and the Grenadines
Samoa	San Marino	Sao Tome and Principe
Saudi Arabia	Senegal	Seychelles
Siachen Glacier	Sierra Leone	Singapore
Sint Maarten	Slovakia	Slovenia
Solomon Islands	Somalia	Somaliland
South Africa	South Georgia and South Sandwich Islands	South Korea
South Sudan	Spain	Sri Lanka
Sudan	Suriname	Swaziland
Sweden	Switzerland	Syria
Taiwan	Tajikistan	Thailand
The Bahamas	Togo	Tonga
Trinidad and Tobago	Tunisia	Turkey
Turkmenistan	Turks and Caicos Islands	Tuvalu
Uganda	Ukraine	United Arab Emirates
United Republic of Tanzania	United States Virgin Islands	Uruguay
Uzbekistan	Vanuatu	Vatican
Venezuela	Vietnam	Wallis and Futuna
West Bank	Western Sahara	Yemen
Zambia	Zimbabwe	
