# Supplementary material for: Tracking Protests Using Geotagged Flickr Photographs
Source: PLoS One. 2016 Mar 1;11(3):e0150466. doi: 10.1371/journal.pone.0150466 (PMC4773018; doi:10.1371/journal.pone.0150466)
Supplement: S2 Table — List of translations of the word “protest” in different languages. (PDF) [file pone.0150466.s002.pdf]

S2 Table. List of translations of the word 'protest' in different languages.

Protest Keyword	Language
احتجاج	Arabic
Protesta	Austrian
Протест	Bulgarian
Protest	Czech
Protest	German
Protest	Estonian
Protesta	Basque
اعتراض	Persian
Protesta	Galician
항의	Korean
Mótmæli	Icelandic
Contestazione	Italian
מחאה	Hebrew
ಪ್ರತಿಭಟನೆ	Kannada
Қарсылық қозғалысы	Kazakh
Protestas	Lithuanian
Протест	Macedonian
Bantahan	Malay
Protest	Dutch
反対運動	Japanese
Protesto	Portuguese
Protest	Romanian
Протест	Russian
Protest	Simple English
Protest	Slovak
Protest	Serbo-Croatian
Protesti	Finnish
Protest	Swedish
[illegible]	Tamil
การประท้วง	Thai
Protesto	Turkish
Протест	Ukranian
פראטעסט	Yiddish
抗議	Chinese
