# Supplementary material for: Tracking Protests Using Geotagged Flickr Photographs
Source: PLoS One. 2016 Mar 1;11(3):e0150466. doi: 10.1371/journal.pone.0150466 (PMC4773018; doi:10.1371/journal.pone.0150466)
Supplement: S3 Table — Total number of photographs per country and region taken and uploaded to Flickr during 2013. (PDF) [file pone.0150466.s003.pdf]

S3 Table. List of the total number of *Flickr* photographs per country and region.

	Country and Region	Photographs
1	Afghanistan	734
2	Aland	436
3	Albania	2,121
4	Algeria	1,419
5	American Samoa	60
6	Andorra	1,577
7	Angola	290
8	Anguilla	76
9	Antarctica	701
10	Antigua and Barbuda	1,065
11	Argentina	49,670
12	Armenia	1,165
13	Aruba	346
14	Ashmore and Cartier Islands	0
15	Australia	324,903
16	Austria	103,291
17	Azerbaijan	1,039
18	Bahrain	2,278
19	Bangladesh	5,679
20	Barbados	678
21	Belarus	5,187
22	Belgium	104,520
23	Belize	887
24	Benin	164
25	Bermuda	524
26	Bhutan	206
27	Bolivia	4,288
28	Bosnia and Herzegovina	2,134
29	Botswana	860
30	Brazil	336,260
31	British Indian Ocean Territory	16
32	British Virgin Islands	123
33	Brunei	2,377
34	Bulgaria	8,580
35	Burkina Faso	292
36	Burundi	197
37	Cambodia	12,672
38	Cameroon	373
39	Canada	404,408
40	Cape Verde	620
41	Cayman Islands	986
42	Central African Republic	91
43	Chad	77
44	Chile	63,386
45	China	149,705
46	Colombia	34,793
47	Comoros	10
48	Cook Islands	40
49	Costa Rica	12,221
50	Croatia	9,979

	Country Name	Pictures
51	Cuba	2,701
52	Curacao	792
53	Cyprus	5,366
54	Czech Republic	65,739
55	Democratic Republic of the Congo	562
56	Denmark	43,290
57	Djibouti	246
58	Dominica	75
59	Dominican Republic	7,477
60	East Timor	329
61	Ecuador	11,058
62	Egypt	12,849
63	El Salvador	2,412
64	Equatorial Guinea	20
65	Eritrea	23
66	Estonia	10,811
67	Ethiopia	1,474
68	Falkland Islands	269
69	Faroe Islands	592
70	Federated States of Micronesia	48
71	Fiji	163
72	Finland	55,050
73	France	493,813
74	French Guiana	880
75	French Polynesia	871
76	French Southern and Antarctic Lands	0
77	Gabon	128
78	Gambia	399
79	Gaza	702
80	Georgia	4,751
81	Germany	494,288
82	Ghana	754
83	Greece	42,367
84	Greenland	484
85	Grenada	84
86	Guam	1,777
87	Guatemala	5,772
88	Guernsey	2,090
89	Guinea	243
90	Guinea Bissau	7
91	Guyana	600
92	Haiti	1,016
93	Heard Island and McDonald Islands	0
94	Honduras	2,082
95	Hong Kong S.A.R.	58,471
96	Hungary	41,437
97	Iceland	15,565
98	India	67,407
99	Indian Ocean Territories	21
100	Indonesia	58,313

	Country Name	Pictures
101	Iran	3,354
102	Iraq	5,505
103	Ireland	94,009
104	Isle of Man	3,874
105	Israel	35,847
106	Italy	492,311
107	Ivory Coast	117
108	Jamaica	1,879
109	Japan	480,205
110	Jersey	2,035
111	Jordan	8,619
112	Kazakhstan	2,757
113	Kenya	4,793
114	Kiribati	7
115	Kosovo	793
116	Kuwait	10,300
117	Kyrgyzstan	836
118	Laos	3,931
119	Latvia	9,534
120	Lebanon	3,243
121	Lesotho	76
122	Liberia	57
123	Libya	577
124	Liechtenstein	292
125	Lithuania	4,706
126	Luxembourg	4,138
127	Macau S.A.R	36
128	Macedonia	1,536
129	Madagascar	1,575
130	Malawi	465
131	Malaysia	65,237
132	Maldives	680
133	Mali	197
134	Malta	4,760
135	Marshall Islands	0
136	Mauritania	220
137	Mauritius	2,188
138	Mexico	118,988
139	Moldova	834
140	Monaco	80
141	Mongolia	1,909
142	Montenegro	2,310
143	Montserrat	37
144	Morocco	10,613
145	Mozambique	1,141
146	Myanmar	5,769
147	Namibia	783
148	Nauru	11
149	Nepal	4,525
150	Netherlands	262,659

	Country Name	Pictures
151	New Caledonia	415
152	New Zealand	62,590
153	Nicaragua	2,319
154	Niger	80
155	Nigeria	552
156	Niue	6
157	Norfolk Island	235
158	North Korea	1,206
159	Northern Cyprus	1,550
160	Northern Mariana Islands	225
161	Norway	64,758
162	Oman	2,935
163	Pakistan	6,258
164	Palau	48
165	Panama	5,693
166	Papua New Guinea	279
167	Paraguay	1,897
168	Peru	21,277
169	Philippines	70,957
170	Pitcairn Islands	2
171	Poland	55,780
172	Portugal	57,766
173	Puerto Rico	8,591
174	Qatar	9,963
175	Republic of Serbia	9,368
176	Republic of the Congo	128
177	Romania	22,819
178	Russia	124,744
179	Rwanda	950
180	Saint Barthelemy	67
181	Saint Helena	170
182	Saint Kitts and Nevis	295
183	Saint Lucia	214
184	Saint Martin	258
185	Saint Pierre and Miquelon	35
186	Saint Vincent and the Grenadines	50
187	Samoa	25
188	San Marino	609
189	Sao Tome and Principe	105
190	Saudi Arabia	17,735
191	Senegal	978
192	Seychelles	261
193	Siachen Glacier	0
194	Sierra Leone	70
195	Singapore	64,376
196	Sint Maarten	1,131
197	Slovakia	13,811
198	Slovenia	8,673
199	Solomon Islands	143
200	Somalia	14

	Country Name	Pictures
201	Somaliland	25
202	South Africa	28,101
203	South Georgia and South Sandwich Islands	33
204	South Korea	121,170
205	South Sudan	60
206	Spain	490,356
207	Sri Lanka	6,128
208	Sudan	950
209	Suriname	444
210	Swaziland	387
211	Sweden	82,758
212	Switzerland	127,250
213	Syria	2,016
214	Taiwan	394,698
215	Tajikistan	191
216	Thailand	90,141
217	The Bahamas	733
218	Togo	116
219	Tonga	28
220	Trinidad and Tobago	2,824
221	Tunisia	3,328
222	Turkey	38,437
223	Turkmenistan	25
224	Turks and Caicos Islands	355
225	Tuvalu	0
226	Uganda	995
227	Ukraine	27,198
228	United Arab Emirates	20,948
229	United Kingdom	1,890,670
230	United Republic of Tanzania	4,822
231	United States of America	3,812,116
232	United States Virgin Islands	1,275
233	Uruguay	6,617
234	Uzbekistan	881
235	Vanuatu	193
236	Vatican	0
237	Venezuela	16,865
238	Vietnam	53,333
239	Wallis and Futuna	1
240	West Bank	8,854
241	Western Sahara	41
242	Yemen	357
243	Zambia	1,311
244	Zimbabwe	478
