# Supplementary material for: Tracking Protests Using Geotagged Flickr Photographs
Source: PLoS One. 2016 Mar 1;11(3):e0150466. doi: 10.1371/journal.pone.0150466 (PMC4773018; doi:10.1371/journal.pone.0150466)
Supplement: S4 Table — Total number of The Guardian articles released during 2013 covering news relating to countries and regions listed. (PDF) [file pone.0150466.s004.pdf]

S4 Table. List of the total number of *The Guardian* news articles per country and region.

	Country and Region	Total
1	Afghanistan	2,212
2	Aland	0
3	Albania	142
4	Algeria	412
5	American Samoa	23
6	Andorra	60
7	Angola	169
8	Anguilla	24
9	Antarctica	268
10	Antigua and Barbuda	8
11	Argentina	1,255
12	Armenia	105
13	Aruba	16
14	Ashmore and Cartier Islands	0
15	Australia	8,430
16	Austria	729
17	Azerbaijan	126
18	Bahrain	322
19	Bangladesh	808
20	Barbados	115
21	Belarus	235
22	Belgium	1,070
23	Belize	86
24	Benin	89
25	Bermuda	120
26	Bhutan	68
27	Bolivia	282
28	Bosnia and Herzegovina	46
29	Botswana	124
30	Brazil	3,076
31	British Indian Ocean Territory	7
32	British Virgin Islands	95
33	Brunei	67
34	Bulgaria	452
35	Burkina Faso	176
36	Burundi	90
37	Cambodia	319
38	Cameroon	208
39	Canada	2,621
40	Cape Verde	55
41	Cayman Islands	94
42	Central African Republic	160
43	Chad	245
44	Chile	687
45	China	5,871
46	Colombia	503
47	Comoros	21
48	Cook Islands	34
49	Costa Rica	274
50	Croatia	438

	Country	Total
51	Cuba	527
52	Curacao	9
53	Cyprus	829
54	Czech Republic	387
55	Democratic Republic of the Congo	215
56	Denmark	928
57	Djibouti	37
58	Dominica	17
59	Dominican Republic	97
60	East Timor	39
61	Ecuador	379
62	Egypt	1,770
63	El Salvador	133
64	Equatorial Guinea	54
65	Eritrea	65
66	Estonia	210
67	Ethiopia	470
68	Falkland Islands	120
69	Faroe Islands	55
70	Federated States of Micronesia	2
71	Fiji	194
72	Finland	613
73	France	7,242
74	French Guiana	24
75	French Polynesia	9
76	French Southern and Antarctic Lands	0
77	Gabon	86
78	Gambia	79
79	Gaza	520
80	Georgia	688
81	Germany	5,569
82	Ghana	546
83	Greece	1,620
84	Greenland	149
85	Grenada	48
86	Guam	42
87	Guatemala	212
88	Guernsey	95
89	Guinea	622
90	Guinea Bissau	37
91	Guyana	61
92	Haiti	270
93	Heard Island and McDonald Islands	2
94	Honduras	188
95	Hong Kong S.A.R.	0
96	Hungary	469
97	Iceland	634
98	India	4,354
99	Indian Ocean Territories	0
100	Indonesia	1,167

	Country	Total
101	Iran	1,873
102	Iraq	2,398
103	Ireland	4,979
104	Isle of Man	136
105	Israel	2,151
106	Italy	3,970
107	Ivory Coast	279
108	Jamaica	378
109	Japan	2,796
110	Jersey	1,419
111	Jordan	1,675
112	Kazakhstan	297
113	Kenya	1,134
114	Kiribati	25
115	Kosovo	169
116	Kuwait	250
117	Kyrgyzstan	69
118	Laos	99
119	Latvia	221
120	Lebanon	641
121	Lesotho	58
122	Liberia	201
123	Libya	885
124	Liechtenstein	77
125	Lithuania	223
126	Luxembourg	378
127	Macau S.A.R	0
128	Macedonia	128
129	Madagascar	144
130	Malawi	209
131	Malaysia	565
132	Maldives	74
133	Mali	790
134	Malta	233
135	Marshall Islands	19
136	Mauritania	71
137	Mauritius	116
138	Mexico	1,743
139	Moldova	210
140	Monaco	501
141	Mongolia	138
142	Montenegro	322
143	Montserrat	26
144	Morocco	317
145	Mozambique	201
146	Myanmar	106
147	Namibia	119
148	Nauru	147
149	Nepal	365
150	Netherlands	1,376

	Country	Total
151	New Caledonia	30
152	New Zealand	2,477
153	Nicaragua	154
154	Niger	249
155	Nigeria	935
156	Niue	6
157	Norfolk Island	4
158	Northern Cyprus	18
159	Northern Mariana Islands	3
160	North Korea	725
161	Norway	1,070
162	Oman	136
163	Pakistan	1,758
164	Palau	40
165	Panama	248
166	Papua New Guinea	296
167	Paraguay	126
168	Peru	442
169	Philippines	816
170	Pitcairn Islands	0
171	Poland	1,155
172	Portugal	1,153
173	Puerto Rico	65
174	Qatar	796
175	Republic of Serbia	0
176	Republic of the Congo	220
177	Romania	660
178	Russia	3,699
179	Rwanda	359
180	Saint Barthelemy	0
181	Saint Helena	6
182	Saint Kitts and Nevis	5
183	Saint Lucia	10
184	Saint Martin	18
185	Saint Pierre and Miquelon	2
186	Saint Vincent and the Grenadines	5
187	Samoa	136
188	San Marino	184
189	Sao Tome and Principe	4
190	Saudi Arabia	749
191	Senegal	254
192	Seychelles	43
193	Siachen Glacier	0
194	Sierra Leone	226
195	Singapore	801
196	Sint Maarten	7
197	Slovakia	194
198	Slovenia	239
199	Solomon Islands	41
200	Somalia	612

	Country	Total
201	Somaliland	43
202	South Africa	2,766
203	South Georgia and South Sandwich Islands	1
204	South Korea	877
205	South Sudan	187
206	Spain	3,877
207	Sri Lanka	641
208	Sudan	414
209	Suriname	34
210	Swaziland	42
211	Sweden	1,467
212	Switzerland	1,216
213	Syria	2,860
214	Taiwan	293
215	Tajikistan	38
216	Thailand	689
217	The Bahamas	87
218	Togo	113
219	Tonga	103
220	Trinidad and Tobago	56
221	Tunisia	338
222	Turkey	1,953
223	Turkmenistan	46
224	Turks and Caicos Islands	22
225	Tuvalu	24
226	Uganda	532
227	Ukraine	747
228	United Arab Emirates	247
229	United Kingdom	29,106
230	United Republic of Tanzania	5
231	United States of America	3,148
232	United States Virgin Islands	0
233	Uruguay	463
234	Uzbekistan	112
235	Vanuatu	31
236	Vatican	632
237	Venezuela	454
238	Vietnam	911
239	Wallis and Futuna	3
240	West Bank	484
241	Western Sahara	20
242	Yemen	467
243	Zambia	249
244	Zimbabwe	413
